# Substance use and mental health outcomes in children and adolescents exposed to household dysfunction: mediating role of emotional intelligence

**DOI:** 10.1192/bjo.2026.12005

**Published:** 2026-06-11

**Authors:** Maite Ramírez, Nerea San Martín, Laia Marques-Feixa, Soledad Romero, José Luís Monteserín-García, Ariadna Mas, Marta Rapado-Castro, María Marín-Vila, Hilario Blasco-Fontecilla, María José Muñoz, Ana González-Pinto, Iñaki Zorrilla, Lourdes Fañanás

**Affiliations:** Psychiatry, Galdakao-Usansolo Hospital, Galdakao, Spain; Department of Neurosciences, University of the Basque Country, Spain; CIBERSAM, Madrid, Spain; IIS Biobizkaia, Cruces, Spain; Evolutionary Biology, Ecology and Environmental Sciences, Universidad de Barcelona Facultad de Biología, Spain; Child and Adolescent Psychiatry and Psychology, Hospital Clinic de Barcelona, Barcelona, Spain; Institut d’Investigacions Biomediques August Pi i Sunyer, Barcelona, Spain; Child and Adolescent Psychiatry, Hospital General Universitario Gregorio Maranon, Madrid, Spain; Psychiatry, University of Melbourne VCCC, Australia; Psychiatry, Puerta de Hierro University Hospital of Majadahonda, Madrid, Spain; Instituto de Investigación, Transferencia e Innovación, Ciencias de la Salud y Escuela de Doctorado, International University of La Rioja, Spain; Adolescent Crisis Unit, Benito Menni Complex Assistencial en Salut Mental, Barcelona, Spain; Psychiatry, Araba University Hospital, Araba, Spain; Bioaraba Health Research Institute, Vitoria, Spain

**Keywords:** Household dysfunction, emotional intelligence, substance use, psychopathological outcomes, child and adolescent population

## Abstract

**Background:**

Household dysfunction represents one of the most prevalent adverse childhood experiences worldwide, and has been previously associated with an increased risk for mental health problems. However, little is known about the protective role of emotional intelligence in this association.

**Aims:**

To explore whether emotional intelligence mediates the relationship between household dysfunction and (a) substance use (age at first use and severity index) and (b) psychopathological status (behavioural/emotional problems, global functioning and number of lifetime psychiatric diagnoses and pharmacological treatments).

**Method:**

A simple mediation model was employed. A total of 187 children and adolescents aged 7–17 years, from the multicentre EPI-Young-Stress Project, were included in the study. Household dysfunction was assessed following the validated Tool for Assessing the Severity of Situations in which Children are Vulnerable (TASSCV) criteria and emotional intelligence was evaluated with the Trait Emotional Intelligence Questionnaire for Children Short Form (TEIQue-CSF) parent version.

**Results:**

There is an indirect-only mediation effect of emotional intelligence on the relationship between household dysfunction and children’s and adolescents’ age at first substance use (proportion mediated: 31.40%, 99% CI −0.53 to −0.02) and severity index (proportion mediated: 23.69%, 99% CI 0.50−0.34), as well as the number of lifetime psychiatric diagnoses (proportion mediated: 45.38%, 99% CI 0.19−0.60) and psychiatric drugs prescribed (proportion mediated: 48.75%, 99% CI 0.19−0.57). A complementary mediation effect of emotional intelligence was found between household dysfunction and emotional/behavioural problems (proportion mediated: 56.37%, 99% CI 0.32−0.80), as well as global functioning (proportion mediated: 54.20%, 99% CI −0.72 to −0.28).

**Conclusions:**

Emotional intelligence emerges as a putative protective factor against the negative consequences of household dysfunction on mental health and substance use. Emotional intelligence should be considered a primary target in preventing and promoting the health of youth exposed to household dysfunction.

Early adversity has been associated with negative health outcomes in the young population.^1^ Among the most potentially pernicious environments in which children and adolescents could grow up, household dysfunction has been described as the most prevalent condition worldwide, with an estimated prevalence of 61.6% in the adolescent population.^2^ Household dysfunction is a complex construct that refers to exposure to different familial stressors that can include both acute and chronic harmful experiences. These may take the form of the abuse of substances, violent or criminal behaviours, and the presence of severe physical or mental disorders among relatives. In this regard, a study focused on household dysfunction conducted by Broekhof et al^2^ found that 22.1% of the sample reported witnessing violence, 18.8% reported parental mental health problems and 7.5% reported parental substance misuse.

Diagnosing children with symptoms related to relational trauma can be particularly challenging, as these children often present with a broad spectrum of symptoms. Research has shown that they may receive multiple diagnoses throughout their lives, including behavioural disorders, personality disorders and anxiety−depressive disorders, with corresponding changes in pharmacological treatments during childhood.^3^ However, the impact of household dysfunction on these severe clinical outcomes, previously linked to relational trauma, remains underexplored. Although the literature is scarce, early exposure to household dysfunction has also been associated with a higher risk of developing emotional and behavioural problems^4^ and a dose–response relationship with internalising and externalising symptoms.^5^ In fact, household dysfunction seems to be a transdiagnostic risk factor for different mental disorders. According to Kessler et al,^6^ clusters of adversities associated with maladaptive family functioning are linked to the highest risk of any mental disorder. This increased risk for psychopathology has also been identified in children living with relatives with substance use disorders, who have a greater tendency to develop other mental disorders.^7^

Exposure to early household dysfunction is believed to disrupt children’s well-being and future health through different pathways, including stress-induced brain changes and less favourable environments for optimal development.^8^ Additionally, household dysfunction may affect and modulate the attachment style and behaviour of children and adolescents, conferring a greater risk of being involved in self-harming actions, such as the use of substances.^9^ Noticeably, the mentioned loop of risk factors may be at the root of future psychopathology. From an emotion regulation perspective, successful adaptation relies on adaptive strategies like cognitive reappraisal,^10^ and meta-analytic evidence positions emotional intelligence as the core set of dispositional skills that facilitate these regulatory processes.^11^ Within resilience frameworks, internal resources like emotional intelligence may counteract the effects of risk. A compelling theoretical rationale exists for examining emotional intelligence as a mediator of household dysfunction’s effects. Chronic stress and disrupted caregiving within dysfunctional households are hypothesised to impede the development of these core emotional skills.^12^ In turn, deficits in emotional intelligence may limit adaptive coping and increase vulnerability to emotional distress, thereby elevating the risk for maladaptive outcomes such as substance use and psychopathology.^13^

In this regard, youths who have had adverse childhood experiences (ACEs) are at a higher risk of early substance use and more severe consumption patterns.^14^ In fact, Kessler et al^6^ concluded that 51–65% of childhood-onset substance use disorders could be attributed to psychosocial early-life adversities. Specifically, experiences of interpersonal trauma appear to be particularly influential in this risk.^15^ Moreover, early substance use has been established as a reliable predictor of a faster transition to both substance use disorders^16^ and other psychiatric disorders.^17^

Although the relationship between household dysfunction and the above-mentioned mental health outcomes has been well documented, less is known about the factors that may tip the scale toward resilience or disease. In that sense, there are some environmental conditions that have been proposed as putative mediators of this association, including familial, community, school social support^18^ and lifetime stressful life events.^19^

In this regard, one of the most important factors believed to confer resilience to ACE is emotional intelligence, defined as the ability to perceive, understand, use and manage emotions. For this reason, individuals with high levels of emotional intelligence may manage their lives more effectively and adapt better to their environment. Emotional intelligence is intrinsically linked to better emotional management, reactivity and regulation, thus being a protective factor for the development of mental health conditions in individuals exposed to stressful situations.^13^ Furthermore, emotional intelligence could act as a mediator between adversity and the emergence of substance use disorders.^20^

The research in this area has traditionally focused on exploring the role of emotional intelligence in children directly exposed to maltreatment or abuse.^21^ In contrast, experiences of household dysfunction have been relatively unexplored. Despite previous studies highlighting that the family environment is essential for children’s emotional regulation development, to the best of our knowledge, this is the first study to specifically evaluate the potential role of emotional intelligence in the association between household dysfunction and these mental health outcomes in children and adolescents. Consequently, the present study aims to explore whether childrens’ and adolescents’ emotional intelligence could mediate the consequences of household dysfunction in two essential dimensions of their health status and well-being: substance use and psychopathological outcomes.

## Method

### Participants

A total of 187 children and adolescents aged 7–17 years, from our multicentre study EPI-Young-Stress Project, were included in the present study. Of these participants, 116 had been diagnosed with a current psychiatric disorder, and 71 were healthy controls. Those with current psychopathology were recruited from six child and adolescent psychiatry units of Spanish hospitals (in-patient clinics, partial hospital admission programmes and out-patient clinics). Children without psychopathology were recruited from advertisements, primary healthcare centres, schools and other community facilities in the same geographical areas. The case and control groups were comparable in terms of age and gender distribution, and were recruited from similar geographical areas and educational stages.

Experienced psychologists and psychiatrists conducted face-to-face interviews to collect information regarding socioeconomic status, maltreatment history, medical/psychiatric history and substance use. These interviews were conducted separately with minors and with their parents or legal guardians. Other sources, such as school or social service reports, were used when needed. Additional information regarding the sample has been reported elsewhere.^3^

### Measures

#### Household dysfunction

A maltreatment history was thoroughly gathered through a semi-structured interview, validated by professionals from the Spanish Social Services, and made available online.^22^ This instrument differentiates among all five types of direct maltreatment: physical abuse, emotional abuse, sexual abuse, physical neglect and emotional neglect. Additionally, the Tool for Assessing the Severity of Situations in which Children are Vulnerable (TASSCV) includes the assessment of other types of maltreatment, such as prenatal maltreatment of the mother, abandonment of the child and household dysfunction. Following the TASSCV criteria, each child maltreatment type was coded as either (a) absent, (b) suspected (if significant signs of neglect or abuse emerged during the evaluation) or (c) confirmed (with clear evidence from social services or family). Suspected and confirmed cases were categorised into the maltreatment group. The severity index was computed by multiplying its severity and frequency scores of the different types of direct maltreatment, which were rated on a four-point Likert scale according to TASSCV criteria. Maltreatment severity was coded according to the characteristics of the experience suffered, as low (1), moderate (2), severe (3) or very severe (4); frequency was coded according to whether child maltreatment had occurred once (1), sometimes (2), often (3) or frequently (4).

Briefly, the concept of household dysfunction refers to circumstances where the domestic environment is deemed to be inappropriate and incompatible with the optimal development of the individual, exhibiting antisocial or deviant patterns. The presence of household dysfunction was defined as the existence of at least one of the following familial conditions: (a) maladaptive behaviour such as aggression or discrimination (including gender-based or intra-family violence); (b) criminal behaviour (theft, drug trafficking or other crimes that cause harm to others); (c) allowing or encouraging the minor’s misuse of drugs; (d) engaging in prostitution in a home in which the minor is present, or proposing prostitution to the minor; and (e) engaging in episodes of drug misuse that are often perceived by the minor.

#### Emotional intelligence

Emotional intelligence was measured using the parent- or guardian-administered Trait Emotional Intelligence Questionnaire for Children Short Form (TEIQue-CSF). This test provides comprehensive coverage of emotion-related facets of children’s personality, including adaptability, addictive tendencies, emotion expression, emotion perception, emotion regulation, low impulsivity, peer relations, self-esteem and self-motivation. The questionnaire comprises 36 short statements on a five-point Likert scale (ranging from 1 = completely disagree, to 5 = agree completely), yielding scores ranging from 36 to 180. The scale has demonstrated good internal consistency in previous research (e.g. Cronbach’s *α* = 0.88).^40^

#### Substance use

The consumption of tobacco, alcohol, cannabis, cocaine, ecstasy and other substances was evaluated in face-to-face interviews, following the Spanish version of the Schedule for Affective Disorders and Schizophrenia for School-Age Children: Present and Lifetime Version DSM-5 (K-SADS-PL-5). Age of substance use initiation was a study variable of interest regardless of the type of drug used. Additionally, an index of severity of substance use was created by multiplying the number of substances used more than once by the frequency of use (once or more per year (1), one or more per month (2), once per week (3), two to three times per week (4), four to six times per week (5) and daily (6)). The result is a continuous variable (between 0 and 30) that measures substance use, considering both the number of different types and frequency of use.

#### Psychopathological outcomes

##### Emotional and behavioural problems

The Achenback’s Child Behavior Checklist (CBCL) was used to evaluate emotional and behavioural problems in the entire sample. The CBCL has well-established psychometric properties, including high internal consistency for its broad band scales.^23^ This parent-report inventory assesses internalising symptoms (anxious/depressed, withdrawn and somatic complaints), externalising symptoms (rule-breaking behaviour and aggressive behaviour) and other symptoms (social problems, thought problems and attention problems), in children aged 6–18 years. For the purpose of this study, a ‘total problems’ score was employed for the whole sample (CBCL-TP), to summarise the aforementioned domains on a continuum spectrum.

##### Number of lifetime psychiatric diagnoses and subtypes of prescribed drugs

Psychopathology was ascertained for the entire sample, using the K-SADS-PL-5.^24^ This is a semi-structured diagnostic interview with demonstrated good to excellent reliability and validity for assessing DSM-5 disorders in children and adolescents. Clinicians reported psychiatric diagnoses for each participant, both currently and throughout the participant’s life, following DSM-5 criteria. All major diagnostic categories were covered and were categorised into the following groups: attention-deficit/hyperactivity disorder, affective disorders, trauma and stress-related disorders, anxiety disorders, behavioural disorders, psychotic disorders and eating disorders. The number of lifetime psychiatric diagnoses was considered as a main variable for the present study. To better characterise the sample, a classification of the current main diagnosis is reported in Table 1.

**Table 1:** Descriptive data of our sample (*N* = 187) Table 1 long description.

Variables	Values
Age, years, mean^a^	13.62 (2.59) [7–17]
Gender^b^	
Female	108 (58%)
Male	79 (42%)
Ethnicity^b^^,^^c^	
European	154 (82%)
Others	33 (18%)
Socioeconomic status^a^	40 (18) [8–66]
Psychiatric diagnosis	
Current psychiatric diagnostic status^b^	
Without a current psychiatric diagnosis	71 (38%)
With a current psychiatric diagnosis	116 (62%)
Primary psychiatric diagnosis dimensions^b^	
Attention-deficit hyperactivity disorder	30 (26%)
Affective disorders	29 (25%)
Trauma/stress-related disorders	19 (16%)
Anxiety disorders	15 (13%)
Behavioural disorders	13 (11%)
Psychotic disorders	7 (6%)
Eating disorders	3 (3%)
Substance use^d^	
At least one substance^b^	107 (57.2%)
Subtypes of substances	
Alcohol^b^	104 (55.6%)
Tobacco^b^	60 (32.1%)
Cannabis^b^	35 (18.7%)
Ecstasy^b^	3 (1.6%)
Cocaine^b^	3 (1.6%)
Others^b^	1 (0.5%)

ADHD, attention-deficit hyperactivity disorder.

a. Values are reported as mean (s.d.) [range].

b. Refers to any use of any of the listed substances. Values are reported as *n* (%).

c. Other ethnicities were Latin American (66%), Maghrebin (16%), sub-Saharan (9%) and others (9%).

d. Data available for *n* = 177.

Furthermore, the pharmacological treatments currently prescribed were categorised into six types (hypnotics/anxiolytics, antidepressants, antipsychotics, mood stabilisers, anticholinergics, psychostimulants and others). The number of prescribed drugs was calculated as the sum of the different types of drugs prescribed daily.

##### Global functioning

The Children’s Global Assessment Scale (CGAS) was used to evaluate the overall functioning and impairment, serving as a complementary instrument to syndrome-specific scales.^25^ The assessment encompasses multiple domains of functioning, including academic performance, social relationships, self-care skills and overall psychological well-being. Specifically, the CGAS was utilised to evaluate children’s lifetime worst global functioning. Scores ranged from 1 to 100, with lower scores indicating a more severe impairment and higher scores indicating better functioning.

### Statistical analysis

Descriptive analyses were performed using IBM SPSS Statistics version 23 for Windows (IBM Corp., Armonk, NY, USA; https://www.ibm.com/products/spss-statistics), and mediation analyses were performed with PROCESS macro version 4.2 for SPSS (Andrew F. Hayes, The Ohio State University, Columbus, OH, USA; https://www.afhayes.com/spss-sas-and-mplus-macros-and-code.html). In alignment with the objectives of this study, a simple mediation model was employed.^26,27^ Only variables exhibiting a substantial correlation with each other were incorporated into the model.

Household dysfunction was used as the explanatory or independent variable (*X*), and emotional intelligence was the mediating variable (*M*). Six dependent variables (*Y*) were successively tested in our approach: (a) age of substance use initiation, (b) substance use severity index, (c) emotional and behavioural problems (CBCL-TP), (d) number of lifetime psychiatric diagnoses, (e) number of different prescribed drugs and (f) global functioning (CGAS score). For a better understanding of the results, the mentioned dependent variables were grouped in two domains: substance use and psychopathological outcomes. For the dichotomous predictor household dysfunction, the partially standardised coefficients reported represent the change in the outcome variable, expressed in standard deviation units, associated with the presence (coded as 1) versus the absence (coded as 0) of household dysfunction.

This statistical approach enables us to study whether the mediation effect of emotional intelligence is indirect-only or complementary. Indirect-only mediation indicates that the direct effect of *X* on *Y* becomes insignificant when *M* is introduced into the model. Conversely, a complementary mediation effect indicates the presence of both direct and indirect effects from *X* to *Y,* and so when *M* is incorporated into the model, statistical significance is maintained, although it can be reduced.

To account for multiple comparisons, the Bonferroni correction was applied across the six mediation models, adjusting the significance threshold to *p* < 0.0083. Accordingly, the statistical significance of the indirect effects was tested using a bootstrapping procedure with 10 000 samples and a bias-corrected 99% confidence interval. The indirect effect was accepted as statistically significant only if its bias-corrected 99% confidence interval excluded zero.^27^ Standardised coefficients for dichotomous *X*, coded by a unit difference, were in a partially standardised form. For dichotomous variables, the interpretation of the partially standardised coefficient centres around the change in the outcome variable (*M* or *Y*), expressed in standard deviations, associated with a one-unit change in the predictor (*X*).

The Cribari–Neto heteroscedasticity-consistent standard error and covariance matrix estimator was employed. For each mediation analysis, the proportion mediated was estimated. This measure estimates the extent to which the pathway accounts for the total effect through the mediating variable.

### Ethics approval and consent to participate

The authors assert that all procedures contributing to this work comply with the ethical standards of the relevant national and institutional committees on human experimentation and with the Helsinki Declaration of 1975, as revised in 2013.

The study received approval from the Ethical Review Board of the participating hospitals, as well as the Bioethics Commission of the University of Barcelona, serving as the coordinating centre (Institutional Review Board approval number: IRB00003099). Families were explicitly informed about the voluntary nature of the study, their rights and the procedures, risks and potential benefits associated with it. Written consent was required from all parents or legal guardians, and the children themselves provided written assent after the nature of the procedure had been fully explained.

## Results

### Variables and sample description

The total sample comprised 187 minors, the majority of whom (82%) were of European origin. There was a slightly greater number of girls (58%). A total of 38% of the sample had no psychiatric diagnosis, whereas 62% had at least one. The most prevalent primary diagnoses were affective disorders and attention-deficit hyperactivity disorder. Further, 44.3% of the sample demonstrated contact with at least one substance. Alcohol was the most commonly used substance, observed in 55.6% of individuals with a history of substance use. See Table 1 for additional information.

A description of the variables included in the mediation analyses is provided in Table 2. According to the TASSCV scale, 27.4% had household dysfunction. See Table 2 for the descriptive statistics of our sample regarding emotional intelligence and the outcomes of interest: (a) age at substance use initiation, (b) substance use severity index, (c) emotional and behavioural problems (CBCL-TP), (d) number of lifetime psychiatric diagnoses, (e) number of different prescribed drugs and (f) global functioning (CGAS).

**Table 2:** Frequencies and means of the clinical or dependent variables in the studied sample of the present study Table 2 long description.

Variables	Frequencies
Household dysfunction (TASSCV)	
Yes	51 (27.4%)
No	135 (72.6%)
	Mean (s.d.) [range]
Emotional intelligence (TEIQue-CSF)	116.37 (1.81) [63–130]
Substances use	
Age at substance use initiation	13.34 (0.21) [5–17]
Substance use severity index	0.93 (1.58) [0–5]
Psychopathological outcomes	
Number of lifetime psychiatric diagnosis	1.62 (0.12) [0–7]
Number of subtypes of prescribed drugs	0.74 (0.98) [0–3]
Emotional and behavioural problems (CBCL-TP)	57.89 (1.22) [36–102]
Global functioning (CGAS)	72.11 (1.61) [10–100]

TASSCV, Tool for Assessing the Severity of Situations in which Children are Vulnerable; TEIQue-CSF, Trait Emotional Intelligence Questionnaire for Children Short Form; CBCL-TP, Achenback’s Child Behavior Checklist - Total Problems score; CGAS, Children’s Global Assessment Scale.

### Mediation analyses results

Household dysfunction was significantly related to all the clinical variables studied. The relationship with substance use was negative with age at onset (*Z* = −3.267; *p* < 0.001) and positive with the severity of use (*Z* = 3.063; *p* = 0.002). The correlation was also significantly positive with the number of psychiatric disorders (*Z* = 5.227; *p* < 0.001), with emotional and behavioural problems (*Z* = 6.027; *p* < 0.001) and with the number of prescribed drugs (*Z* = 4.293; *p* < 0.001). Conversely, a negative correlation was observed with global functioning (*Z* = −5.718; *p* < 0.001). We also examined the possibility of competitive mediation (suppression), which occurs when direct and indirect effects have opposite signs. No evidence of this pattern was found in our models; all significant direct and indirect paths were consistent in direction.

#### Substance use

**Fig. 1:**
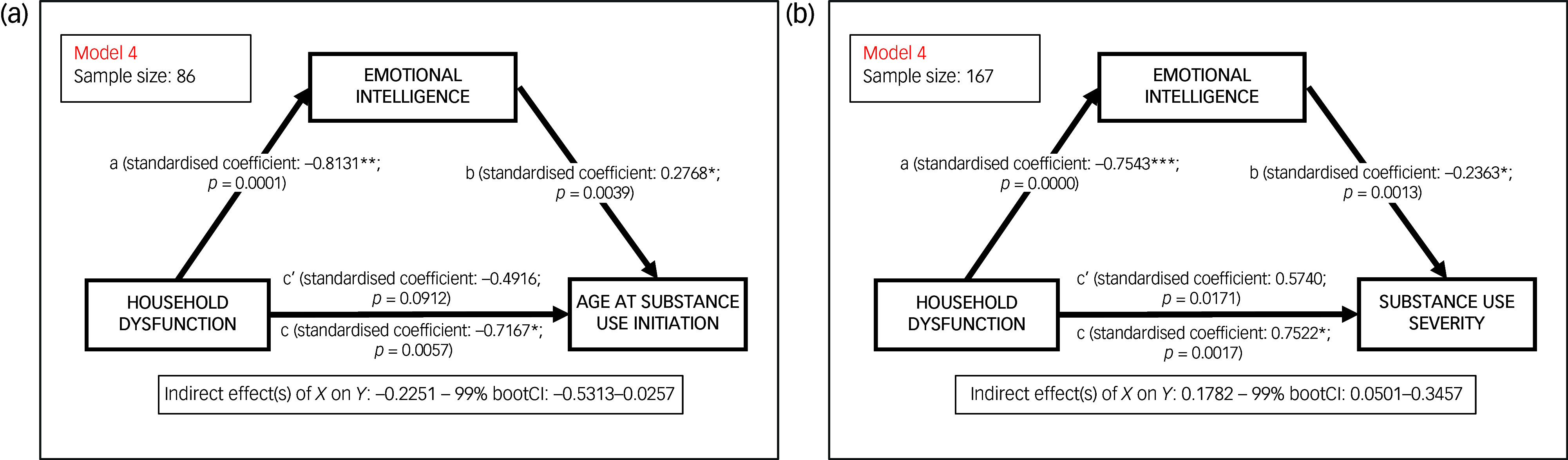
Fig. 1 long description. Simple mediation model of emotional intelligence in the relationship between household dysfunction and substance use outcomes. (a) Age at substance use onset. (b) Substance use severity index. Standardised path coefficients and significance levels (indicated with asterisks) are shown on each arrow. *p < 0.0083, **p < 0.0001, ***p < 0.00001.

Results of the mediation analyses, including household dysfunction, emotional intelligence and outcomes related to substance use, are depicted in Fig. 1.

##### Age at substance use initiation

There is an indirect-only mediation effect of emotional intelligence on the age at onset of substance use, since the effect of household dysfunction on the age at onset of substance use is no longer significant when emotional intelligence is introduced into the model (see Fig. 1(a)). When household dysfunction is present, emotional intelligence decreases. Additionally, as emotional intelligence decreases, the age at substance use initiation is also reduced. Thus, emotional intelligence exerts an opposite-direction effect, suppressing the effect of household dysfunction on substance use onset age. The percentage of the age at onset of substance use that is related to both household dysfunction and emotional intelligence was 17.32% (*R* ^2^). The proportion mediated, or the proportion of the total effect explained by the pathway through the mediating variable, was 31.40%.

##### Substance use severity

There is an indirect-only mediation effect of emotional intelligence between household dysfunction and substance use severity. Thus, the influence of household dysfunction on substance use severity becomes non-significant when emotional intelligence is incorporated into the model, as illustrated in Fig. 1(b). Therefore, emotional intelligence exerts an opposite-direction effect, suppressing the negative effect of household dysfunction. The proportion of the severity of substance use variable that is attributable to the household dysfunction and emotional intelligence combined was 15.54% (*R* ^2^), and the proportion mediated was 23.69%.

#### Psychopathological outcomes

**Fig. 2:**
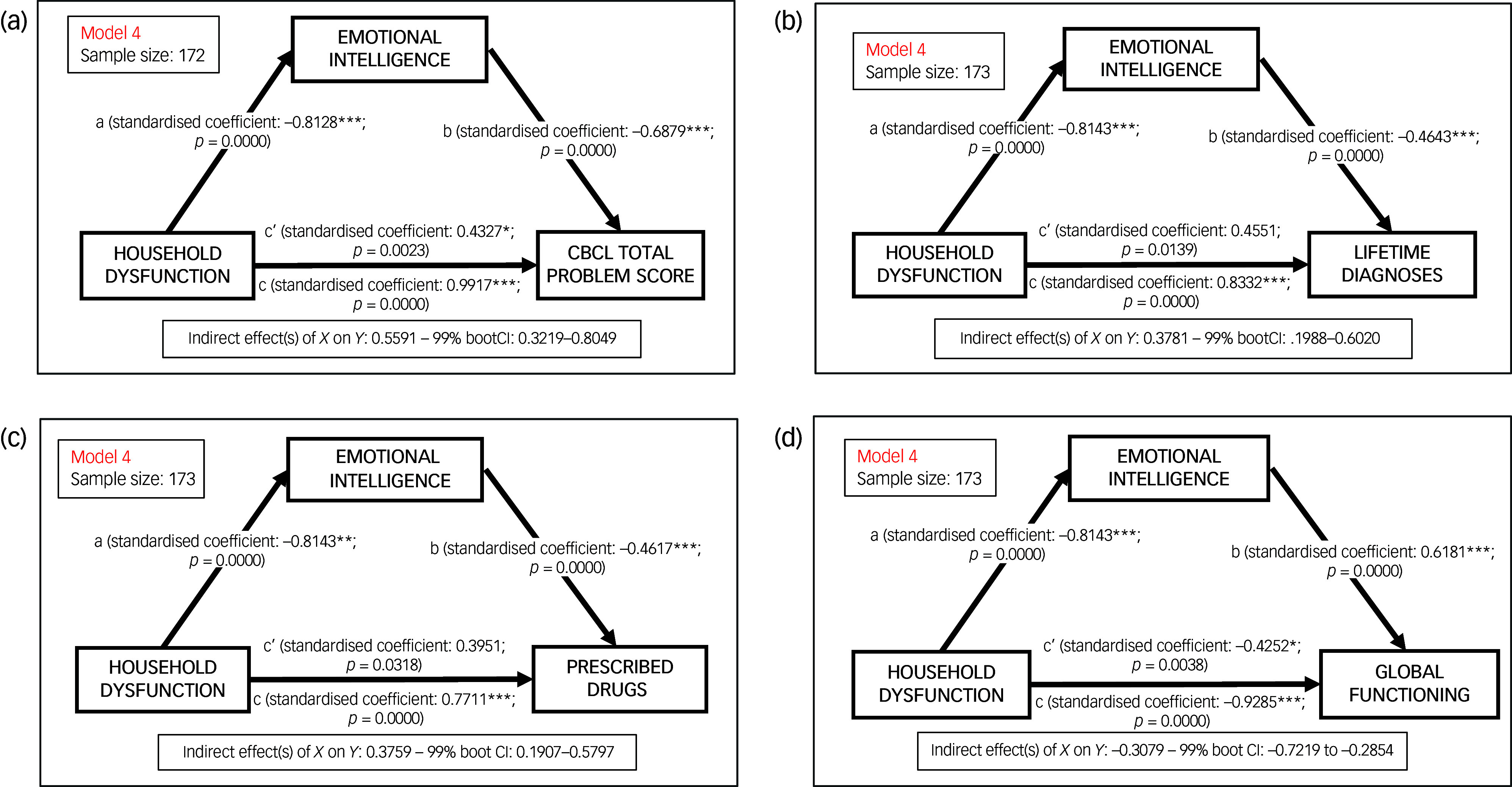
Simple mediation model of emotional intelligence in the relationship between household dysfunction and clinical/functional outcomes. (a) Emotional/behavioural problems (Achenback’s Child Behavior Checklist (CBCL) Total Problems score). (b) Number of lifetime psychiatric diagnoses. (c) Number of prescribed psychiatric drugs. (d) Global functioning (Children’s Global Assessment Scale score). Path coefficients and statistical significance are displayed directly in the figure. *p < 0.0083, **p < 0.0001, ***p < 0.00001.

Results of the mediation analyses, including household dysfunction, emotional intelligence and outcomes related to psychopathological outcomes, are depicted in Fig. 2.

##### Emotional and behavioural problems (CBCL-TP)

There is a complementary mediation of emotional intelligence between household dysfunction and the CBCL-TP score (see Fig. 2(a)). Thus, the influence of household dysfunction on emotional and behavioural problems remains significant when introducing emotional intelligence into the model, although the effect size reduces. Household dysfunction decreases emotional intelligence, which is associated with increases in the CBCL-TP score. The proportion of the CBCL-TP attributable to the household dysfunction and emotional intelligence combined was 60.54% (*R* ^2^), and the proportion mediated was 56.37%.

##### Number of lifetime psychiatric diagnoses

There is an indirect-only mediation effect of emotional intelligence between household dysfunction and the number of different lifetime psychiatric diagnoses received. Consequently, when emotional intelligence is incorporated into the model, the impact of household dysfunction on the number of lifetime psychiatric diagnoses is suppressed, as seen in Fig. 2(b). Specifically, a negative correlation was observed between emotional intelligence and the number of psychiatric diagnoses. The proportion of the number of psychiatric diagnoses variable attributable to household dysfunction and emotional intelligence combined was 37.12% (*R* ^2^), and the proportion mediated was 39.46%.

##### Number of drugs prescribed (polypharmacy)

There is an indirect-only mediation effect of emotional intelligence between household dysfunction and the number of drugs prescribed to psychiatric patients, since the influence of household dysfunction ceases to be significant when emotional intelligence is added to the model (see Fig. 2(c)). As emotional intelligence decreases, the quantity of prescribed psychiatric drugs increases. The proportion of the number of different types of prescribed drugs that are attributable to household dysfunction and emotional intelligence combined was 30.21% (*R* ^2^), and the proportion mediated was 48.75%.

##### Global functioning (CGAS)

A complementary mediation effect of emotional intelligence was identified between household dysfunction and the lifetime worst CGAS score, since the influence of household dysfunction on CGAS remained significant when emotional intelligence was incorporated into the model, but the effect size was diminished (see Fig. 2(d)). Specifically, our results indicate that, as emotional intelligence decreases, the global functioning of the participants also decreases. The proportion of the global functioning variable attributable to household dysfunction and emotional intelligence combined was 50.16% (*R* ^2^), and the proportion mediated was 54.20%.

All indirect effects remained statistically significant even under these stricter criteria, further supporting the robustness of the proposed model.

## Discussion

As expected, our findings indicate that children exposed to elevated household dysfunction tend to present with lower levels of emotional intelligence. This underscores the importance of a stable and functional family environment in fostering the emotional competence necessary for healthy development.

Interestingly, in our study, emotional intelligence emerges as a putative protective factor against the negative consequences that household dysfunction has on youths’ risk for substance use, emotional and behavioural problems, and clinical psychiatric conditions. Actually, our results indicate that emotional intelligence has a protective effect against all the explored outcomes.

First, regarding substance use, our findings suggest that children and adolescents exposed to dysfunctional households are at a greater risk of consuming substances at earlier stages and with a higher severity. This is in great accordance with Broekhof et al,^2^ who report that children exposed to household dysfunction present a higher lifetime risk of substance use. Furthermore, other researchers have described an earlier onset and a higher severity of substance use among people exposed to ACE and recurrent trauma.^15^ Unfortunately, these authors do not explore emotional intelligence as a mediator in this association. Our data, however, clearly detected the protective effect of emotional intelligence on the age at initiation of substance use, where we found an indirect-only mediation effect of emotional intelligence and a proportion mediated of 31.40%, showing that emotional intelligence accounts for nearly a third of the effect of household dysfunction on substance use onset. This suggests that children exposed to household dysfunction and low emotional intelligence may be more likely to engage in substance use, possibly as a means of managing or regulating their negative emotions. However, the findings of this study demonstrate that if emotional intelligence is fostered, even in the presence of household dysfunction, the onset of substance use may not be altered, thereby acting as a clear protective factor for substance use onset. According to our results, emotional intelligence also has a protective effect on the severity of substance use. Although the proportion mediated is somewhat smaller (approximately 24%), it remains substantial. It indicates that a considerable part of the more severe and frequent use pattern associated with household dysfunction is explained by lower emotional intelligence. These results are in line with former research indicating that emotional intelligence plays a key role in the development of substance use and behavioural addictions.^31^ Since emotional intelligence appears to be related to impulse control and stress management, it is not surprising that it exerts a pivotal influence on early-onset substance use and severity.

Concerning emotional and behavioural outcomes, our study points out that youths exposed to household dysfunction present a higher risk of developing behavioural and emotional problems. According to our findings, the research by Holmes et al^5^ evidences that household dysfunction experiences mediate over half (56%) of household dysfunction’s effect on both externalising and internalising behaviours, a relationship that has already been well described for other ACEs. Furthermore, two recent studies claimed that household dysfunction predicts higher rates of deviant behaviours such as illicit drug use or stealing.^4^ In this regard, a recent study highlights that the co-occurrence of ACE predicts a higher risk of maladaptive behaviours, including the commitment of felonies and a higher risk of juvenile arrest.^32^ However, the authors did not discriminate between abuse and household dysfunction. Interestingly, household dysfunction is believed to affect youths’ future global functioning by means of the mentioned behavioural deviations, which have been, in turn, associated with reduced childhood well-being, poorer academic performance and later mental health outcomes.^35^

Regarding the role of emotional intelligence, our findings suggest that emotional intelligence acts as a protective factor against the aforementioned behavioural and emotional deviations. This protective relationship has been previously highlighted in the literature, with higher levels of emotional intelligence reducing adolescents’ involvement in risky behaviours. Additionally, different dimensions of emotional intelligence have been negatively associated with internalising and externalising behaviours, with self-control being a predictor of lower externalising problems and well-being a predictor of lower internalising problems.^3^

Second, regarding mental health outcomes, our study demonstrates the existence of an indirect-only mediation effect between household dysfunction and the number of lifetime psychiatric diagnoses received and global functioning, with a proportion mediated of nearly 40%. In this regard, the relationship between ACEs and adverse mental health outcomes has been extensively reported in the literature, both for direct forms of maltreatment^3^ and household dysfunction.^33^ Furthermore, our findings suggest an indirect-only mediation effect between household dysfunction and the number of pharmacological treatments prescribed, showing that nearly half of household dysfunction’s effect on polypharmacy could operate through emotional intelligence. Actually, ACEs have been clearly associated with both a higher risk of mental disorders and a worse clinical prognosis.^34^ However, the evidence of a worse prognosis among individuals solely exposed to household dysfunction is scarce, although a previous study identified a poorer adjustment among individuals living in suboptimal familial environments.^35^

The *R* ^2^ values indicate that the combined effect of household dysfunction and emotional intelligence explains a meaningful share of the variability in key outcomes – from about a sixth of the severity in substance use to nearly two-thirds of the variance in broad emotional/behavioural problems. This reinforces that, although other factors are involved, targeting the familial environment and emotional skills (as captured by our model) could address a core component of risk for these conditions in youth.

Finally, the fact that emotional intelligence acts as a mediator of the number of psychiatric diagnoses and the clinical prognosis is not surprising, considering previous evidence supporting emotional intelligence as a protective factor for mental health problems in adolescents.^13^ Indeed, classical studies determined that emotional intelligence moderates the relationship between stress and mental health, since individuals with higher levels of emotional intelligence can mitigate the harmful effects of adverse situations and diminish the levels of perceived emotional distress.^36^ Moreover, according to our findings, emotional intelligence enhances psychological adjustment in adolescents. In this regard, emotional intelligence is believed to contribute to personal resilience after stress, since it is related to a more optimal emotional reactivity and less emotional dysregulation, two factors that have been observed to be underlying transdiagnostic mental disorders.^37^ In fact, emotional intelligence is associated with better use, understanding and management of emotions, which leads to healthy choices for personal growth and well-being. Indeed, the indirect-only mediating effect found between household dysfunction and pharmacological treatments suggests that increasing emotional intelligence in children exposed to household dysfunction could help reduce the psychiatric polypharmacy associated with them.

### Limitations

The present study has some limitations. First, it is highly probable that household dysfunction coexists with other subtypes of childhood maltreatment. Moreover, employing a dichotomous variable of household dysfunction does not allow us to explore the potential effects of the severity and accumulation of different experiences, included in this construct. In fact, this study defined household dysfunction as the presence of severe familial conditions, including episodes of substance use, maladaptive and violent behaviours, or prostitution witnessed by minors. It should be noted that the definition of household dysfunction varies across studies, with other authors including additional experiences such as parental divorce.

Additionally, the sample size of 187 youth is modest for a multiple mediation model that includes six dependent variables and various covariates. Nevertheless, the results remained significant even after applying the Bonferroni correction.

Concerning the possibility of reverse causation, such as psychiatric symptoms affecting emotional intelligence, this cannot be completely dismissed in a cross-sectional design. Nevertheless, the directionality outlined in the model – from household dysfunction to emotional intelligence and, subsequently, to various outcomes – is rooted in well-established theoretical frameworks and bolstered by other longitudinal studies.^28–30^ Therefore, the current findings align with a theoretically and clinically valid pathway, offering preliminary evidence that justifies further exploration in prospective longitudinal studies.

The generalisability of our findings may be limited by the specific cultural and healthcare context of Spain and the predominantly European origin (82%) of our sample. Future studies in more diverse cultural and ethnic populations are needed.

In conclusion, household dysfunction is a form of early-life stress that increases the risk of youths’ mental health conditions, including the onset and severity of substance use, emotional and behavioural problems, and clinical psychiatric outcomes such as comorbidity and polypharmacy. In our study, emotional intelligence appears to be a protective factor against the negative consequences of household dysfunction. The findings of this study suggest several potential implications for practice and policy, which warrant consideration for future research and implementation efforts. First, regarding prevention, our results underscore the potential value of enhancing early identification of household dysfunction in settings frequented by children, such as primary care and schools. Incorporating brief, non-stigmatising screenings for familial adversity into routine assessments could facilitate earlier connection to support services, potentially mitigating risk before more severe outcomes emerge. Second, concerning intervention, the consistent mediating role of emotional intelligence suggests that interventions designed to bolster these skills may be particularly beneficial for youth exposed to household dysfunction. Existing evidence-based programmes, such as the school-wide Social and Emotional Learning (SEL) curricula (e.g. the RULER programme (recognizing, understanding, labeling, expressing, regulating) developed by the Yale Center for Emotional Intelligence^38^), provide a viable model for universal promotion of emotional competencies. For youth showing early signs of difficulty, integrating emotional intelligence-focused modules into therapeutic approaches could directly target the psychological mechanism identified here. Third, at the policy level, public health and educational strategies could benefit from a greater integration of emotional skills promotion. This includes supporting the implementation of SEL programmes and training for child-facing professionals to recognise adversity and its emotional correlates.^39^ Positioning emotional competence as a developmental asset within health and education policy frameworks represents a promising, proactive approach to building resilience.

## Data Availability

The data that support the findings of this study are available from the corresponding author, A.G.-P., upon reasonable request.

## Table 1 Long description

The table presents descriptive data for a sample of 187 individuals. It includes variables such as age, gender, ethnicity, socioeconomic status and psychiatric diagnoses. The mean age is 13.62 years with a standard deviation of 2.59, ranging from 7 to 17 years. Gender distribution shows 108 females (58 percent) and 79 males (42 percent). Ethnicity is divided into European (154 individuals, 82 percent) and others (33 individuals, 18 percent). Socioeconomic status has a mean value of 40 with a standard deviation of 18, ranging from 8 to 66. Psychiatric diagnostic status indicates 71 individuals (38 percent) without a current psychiatric diagnosis and 116 individuals (62 percent) with a current psychiatric diagnosis. Primary psychiatric diagnosis dimensions include attention-deficit hyperactivity disorder (30 individuals, 26 percent), affective disorders (29 individuals, 25 percent), trauma and stress-related disorders (19 individuals, 16 percent), anxiety disorders (15 individuals, 13 percent), behavioral disorders (13 individuals, 11 percent), psychotic disorders (7 individuals, 6 percent), and eating disorders (3 individuals, 3 percent). Substance use is reported by 107 individuals (57.2 percent), with subtypes including alcohol (104 individuals, 55.6 percent), tobacco (60 individuals, 32.1 percent), cannabis (35 individuals, 18.7 percent), ecstasy (3 individuals, 1.6 percent), cocaine (3 individuals, 1.6 percent) and others (1 individual, 0.5 percent).

Navigate back to Table 1.

## Table 2 Long description

The table presents data on various clinical variables in a studied sample. It includes two columns: Variables and Frequencies. The Variables column lists categories such as household dysfunction, emotional intelligence, substances use and psychopathological outcomes. The Frequencies column provides corresponding data, including percentages, means, standard deviations, and ranges. Household dysfunction is reported in 27.4% of cases. Emotional intelligence has a mean score of 116.37 with a standard deviation of 18.1 and a range from 63 to 130. Substances use details include age at initiation, substance use severity index and various psychopathological outcomes such as the number of lifetime psychiatric diagnoses, number of prescribed drugs, emotional and behavioral problems, and global functioning scores.

Navigate back to Table 2.

## Fig. 1 Long description

A diagram illustrating the relationship between household dysfunction, emotional intelligence, and substance use outcomes. Panel A: A mediation model showing the relationship between household dysfunction and age at substance use onset, mediated by emotional intelligence. The model includes standardized coefficients and significance levels on each arrow. Household dysfunction negatively affects emotional intelligence, which in turn affects the age at substance use onset. Panel B: A mediation model showing the relationship between household dysfunction and substance use severity index, mediated by emotional intelligence. The model includes standardized coefficients and significance levels on each arrow. Household dysfunction negatively affects emotional intelligence, which in turn affects the substance use severity index.

Navigate back to Fig. 1.

## References

[1] Felitti VJ , Anda RF , Nordenberg D , Williamson DF , Spitz AM , Edwards V , et al. Relationship of childhood abuse and household dysfunction to many of the leading causes of death in adults: The Adverse Childhood Experiences (ACE) Study. Am J Prev Med 1998; 14: 245–58. 9635069 10.1016/s0749-3797(98)00017-8

[2] Broekhof R , Nordahl HM , Bjørnelv S , Selvik SG. Prevalence of adverse childhood experiences and their co-occurrence in a large population of adolescents: a Young HUNT 3 study. Soc Psychiatry Psychiatr Epidemiol 2022; 57: 2359–66. 35460058 10.1007/s00127-022-02277-z PMC9672007

[3] Marques-Feixa L , Palma-Gudiel H , Romero S , Moya-Higueras J , Rapado-Castro M , Castro-Quintas Á , et al. Childhood maltreatment disrupts HPA-axis activity under basal and stress conditions in a dose–response relationship in children and adolescents. Psychol Med 2023; 53: 1060–73. 34269169 10.1017/S003329172100249X PMC9976019

[4] Bussemakers C , Kraaykamp G , Schoon I , Tolsma J. Household dysfunction and child development: Do financial resources matter? Adv Life Course Res 2022; 51: 100447. 36652310 10.1016/j.alcr.2021.100447

[5] Holmes H , Darmanthe N , Tee K , Goodchild M. Adverse childhood experiences–household stressors and children’s mental health: a single centre retrospective review. BMJ Paediatr Open 2021; 5: e001209. 10.1136/bmjpo-2021-001209 PMC8372878 34485707

[6] Kessler RC , McLaughlin KA , Green JG , Gruber MJ , Sampson NA , Zaslavsky AM , et al. Childhood adversities and adult psychopathology in the WHO World Mental Health Surveys. Br J Psychiatry 2010; 197: 378–85. 21037215 10.1192/bjp.bp.110.080499 PMC2966503

[7] Lander L , Howsare J , Byrne M. The impact of substance use disorders on families and children: from theory to practice. Soc Work Public Health 2013; 28: 194–205. 23731414 10.1080/19371918.2013.759005 PMC3725219

[8] Shonkoff JP , Garner AS , Siegel BS , Dobbins MI , Earls MF , Garner AS , et al. The lifelong effects of early childhood adversity and toxic stress. Pediatrics 2012; 129: e232–46. 22201156 10.1542/peds.2011-2663

[9] Craig JM , Wolff KT , Pierce K , Zettler H , Baglivio MT. Childhood abuse, neglect, household dysfunction, and juvenile recidivism: the mediating role of social bonds. J Crim Justice 2022; 82: 101998.

[10] Gross JJ. Emotion regulation: current status and future prospects. Psychol Inq 2015; 26: 1–26.

[11] Salovey P , Mayer JD. Emotional intelligence. Imagin Cogn Pers 1990; 9: 185–211.

[12] Barragán Martín AB , del Molero Jurado MM , del Pérez-Fuentes MC , Oropesa Ruiz NF , Martos Martínez Á , del Són Márquez M , et al. Interpersonal support, emotional intelligence and family function in adolescence. Int J Environ Res Public Health 2021; 18: 5145. 34066285 10.3390/ijerph18105145 PMC8152060

[13] Davis SK , Humphrey N. Emotional intelligence as a moderator of stressor–mental health relations in adolescence: evidence for specificity. Pers Individ Differ 2012; 52: 100–5.

[14] Leza L , Siria S , López-Goñi JJ , Fernández-Montalvo J. Adverse childhood experiences (ACEs) and substance use disorder (SUD): a scoping review. Drug Alcohol Depend 2021; 221: 108563. 33561668 10.1016/j.drugalcdep.2021.108563

[15] Aas M , Sideli L , Franceschini C , Alameda L , Trotta G , Coco GL , et al. The role of interpersonal trauma and substance use in mental health: a large population-based study. Psychiatry Res 2024; 333: 115712. 38219350 10.1016/j.psychres.2023.115712 PMC11137873

[16] Degenhardt L , Stockings E , Patton G , Hall WD , Lynskey M. The increasing global health priority of substance use in young people. Lancet Psychiatry 2016; 3: 251–64. 26905480 10.1016/S2215-0366(15)00508-8

[17] Brownlie E , Beitchman JH , Chaim G , Wolfe DA , Rush B , Henderson J. Early adolescent substance use and mental health problems and service utilisation in a school-based sample. Can J Psychiatry 2019; 64: 116–25. 29929386 10.1177/0706743718784935 PMC6405806

[18] McCoy K , Tibbs JJ , DeKraai M , Hansen DJ. Household dysfunction and adolescent substance use: moderating effects of family, community, and school support. J Child Adolesc Subst Abuse 2020; 29: 68–79.

[19] White HR , Widom CS. Three potential mediators of the effects of child abuse and neglect on adulthood substance use among women. J Stud Alcohol Drugs 2008; 69: 337–47. 18432375 10.15288/jsad.2008.69.337

[20] Edalati H , Krank MD. Childhood maltreatment and development of substance use disorders: a review and a model of cognitive pathways. Trauma Violence Abuse 2016; 17: 454–67. 25964275 10.1177/1524838015584370

[21] Peng P , Zhang Z , Wang W , Lee K , Wang T , Wang C , et al. A meta-analytic review of cognition and reading difficulties: individual differences, moderation, and language mediation mechanisms. Psychol Bull 2022; 148: 227–72.

[22] Servicio de Atención Primaria y Especializada de la Región de Murcia. *Tool for Assessing the Severity of Situations in which Children are Vulnerable (TASSCV)*. Comunidad Autónoma de la Región de Murcia, 2012 (https://dspace.carm.es/jspui/bitstream/20.500.11914/1243/1/4381-Texto%20Completo%201%20Instrumento%20para%20la%20valoraci%C3%B3n%20de%20la%20gravedad%20de%20las%20situaciones%20de%20desprotecci%C3%B3n%20infantil%20%281%29.pdf).

[23] Achenbach TM , Rescorla LA. Manual for the ASEBA School-Age Forms & Profiles. University of Vermont, Research Center for Children, Youth, & Families, 2001.

[24] Kaufman J , Birmaher B , Brent D , Rao U , Flynn C , Moreci P , et al. Schedule for affective disorders and schizophrenia for school-age children-present and lifetime version (K-SADS-PL): initial reliability and validity data. J Am Acad Child Adolesc Psychiatry 1997; 36: 980–8. 9204677 10.1097/00004583-199707000-00021

[25] Shaffer D , Gould MS , Brasic J , Ambrosini P , Fisher P , Bird H , et al. A childrens global assessment scale (CGAS). Arch Gen Psychiatry 1983; 40: 1228–31. 6639293 10.1001/archpsyc.1983.01790100074010

[26] Zhao X , Lynch JG Jr , Chen Q. Reconsidering Baron and Kenny: myths and truths about mediation analysis. J Consum Res 2010; 37: 197–206.

[27] Hayes AF. Introduction to Mediation, Moderation, and Conditional Process Analysis, Second Edition: A Regression-Based Approach. Guilford Publications, 2017.

[28] Danese A , Widom CS. Associations between objective and subjective experiences of childhood maltreatment and the course of emotional disorders in adulthood. JAMA Psychiatry 2023; 80: 1009–16. 37405795 10.1001/jamapsychiatry.2023.2140 PMC10323762

[29] Kim-Spoon J , Brieant A , Folker A , Lindenmuth M , Lee J , Casas B , et al. Psychopathology as long-term sequelae of maltreatment and socioeconomic disadvantage: neurocognitive development perspectives. Dev Psychopathol 2024; 36: 2421–32. 38476054 10.1017/S0954579424000531 PMC11393179

[30] Nweze T , Ezenwa M , Ajaelu C , Okoye C. Childhood mental health difficulties mediate the long-term association between early-life adversity at age 3 and poorer cognitive functioning at ages 11 and 14. J Child Psychol Psychiatry 2023; 64: 952–65. 36751886 10.1111/jcpp.13757

[31] Henning C , Crane AG , Taylor R , Parker J. Emotional intelligence: relevance and implications for addiction. Curr Addict Rep 2021; 8: 28–36.

[32] Giovanelli A , Mondi CF , Reynolds AJ , Ou SR. Adverse childhood experiences: mechanisms of risk and resilience in a longitudinal urban cohort. Dev Psychopathol 2020; 32: 1418–39. 31663487 10.1017/S095457941900138X PMC7190431

[33] Negriff S. ACEs are not equal: examining the relative impact of household dysfunction versus childhood maltreatment on mental health in adolescence. Soc Sci Med 2020; 245: 112696. 31785426 10.1016/j.socscimed.2019.112696 PMC6961803

[34] Lippard ETC , Nemeroff CB. The devastating clinical consequences of child abuse and neglect: increased disease vulnerability and poor treatment response in mood disorders. Am J Psychiatry 2020; 177: 20–36. 31537091 10.1176/appi.ajp.2019.19010020 PMC6939135

[35] Higgins DJ , McCabe MP. Maltreatment and family dysfunction in childhood and the subsequent adjustment of children and adults. J Fam Violence 2003; 18: 107–20.

[36] Gohm C , Corser G , Dalsky D. Emotional intelligence under stress: useful, unnecessary, or irrelevant? Pers Individ Differ 2005; 39: 1017–28.

[37] Reyes-Wapano MR. Literature review: gender, parenting style and temperament influence the development of emotional intelligence. Int J Res Innov Soc Sci 2021; 05: 595–605.

[38] Brackett MA , Rivers SE , Reyes MR , Salovey P. Enhancing academic performance and social and emotional competence with the RULER feeling words curriculum. Learn Individ Differ 2012; 22: 218–24.

[39] Durlak JA , Weissberg RP , Dymnicki AB , Taylor RD , Schellinger KB. The impact of enhancing students social and emotional learning: a meta-analysis of school-based universal interventions. Child Dev 2011; 82: 405–32. 21291449 10.1111/j.1467-8624.2010.01564.x

[40] Petrides KV , Furnham A. Trait Emotional Intelligence Questionnaire (TEIQue). Technical Manual. London Psychometric Laboratory, 2009.

